# Combination of Levamisole with Prednisone in Treating Recurrent Major Aphthous Ulcer in a Young Boy: A Case Report

**DOI:** 10.3390/clinpract11020038

**Published:** 2021-05-06

**Authors:** Jyoti Prajapat, Rajesh Prajapat, Sanjeev B. Khanagar, Satish Vishwanathaiah, Sachin Naik, Chitra Jhugroo, Darshan Devang Divakar

**Affiliations:** 1 Private Dental Practitioner, Oral Medicine and Radiology, Gurugram 122001, India; 2 Private Practitioner, Prosthodontics Crown and Bridge, Al Jazeera Medical Complex, Al Naseem, Riyadh 14236, Saudi Arabia; drrajeshprajapat10@gmail.com; 3 Preventive Dental Science Department, College of Dentistry, King Saud Bin Abdulaziz University for Health Sciences, Riyadh 11426, Saudi Arabia; sanjeev.khanagar76@gmail.com; 4 King Abdullah International Medical Research Center, Riyadh 11426, Saudi Arabia; 5 Division of Pedodontics, Department of Preventive Dental Sciences, College of Dentistry, Jazan University, Jazan 44512, Saudi Arabia; drsatish77@gmail.com; 6 Dental Biomaterials Research Chair, Dental Health Department, College of Applied Medical Sciences, King Saud University, P. O. Box 10219, Riyadh 11433, Saudi Arabia; snaik@ksu.edu.sa (S.N.); chitra.1809@yahoo.com (C.J.); darshandevang@gmail.com (D.D.D.); 7 Nano Dental Clinic, Vacoas 74101, Mauritius; 8 Department of Oral Medicine and Radiology, Faculty of Dentistry, Levy Mwanawasa Medical University (LMMU), Ministry of Health, Lusaka 10101, Zambia

**Keywords:** major aphthous ulcers, canker sores, immunomodulators, corticosteroids, levamisole, visual analogue scale

## Abstract

Recurrent aphthous stomatitis (RAS) is an oral condition characterized by painful oral ulcerations. While the clinical features of this disease are easily defined, the etiology remains unclear. Thus, existing treatments are still unsatisfactory in reducing the severity, healing, and recurrence rate; however, there is no permanent and definitive treatment. Effective treatment for aphthous stomatitis is not available, and those treatments available mainly focus on suppressing its symptoms. We are reporting a case of a 17-year-old boy who presented with a 3-year history of multiple recurrent major ulcers in the oral cavity. Levamisole with steroids has been used in many clinical trials to treat aphthous ulcers, showing an improvement in pain, discomfort, healing time, and reduction in the number of ulcers. The same method was used to treat our patient, who showed promising results, with no recurrence for one year. Levamisole is a safe, easily tolerable and promising drug for the treatment of RAS.

## 1. Introduction

Recurrent aphthous stomatitis (RAS) is an oral mucosal lesion characterized by multiple, recurrent, oval-shaped ulcers, where the ulcer floor is covered with yellowish pseudo-membrane and surrounded by an erythematous halo. These ulcers affect up to 25% of the general population, and the 3-month recurrence rate is about 50%. Females are more commonly affected than males [1]. It occurs most commonly in the 1st to 4th decades of life, and in individuals of higher socioeconomic status [2]. Minor RAS comprises between 70% and 87% of all RAS forms, and a recent study places its frequency rate at 17.7% in the general population. The remaining 7–10% of cases are of either the major or herpetiform types. The treatment for all cases is symptomatic, and the principal aim of treatment is to decrease inflammation and pain relief by administering topical or systemic treatments. One of the agents used for the systemic treatment of RAS is levamisole, due to its various immunological effects. Levamisole normalizes the phagocytic activity of macrophages and neutrophils, regulates T-cell activity, and modulates the activity of human interferons (IFNs) and the serum levels of interleukin IL-6 and IL-8. In the RAU cases, it helps in the normalization of the CD4+/CD8+ cell ratio, and increases the levels of serum immunoglobulin A (IgA) and IgM [3].

Cases of recurrent major aphthous ulcers that show severe pain symptoms, dysphagia, and fever typically require systemic therapy. There is a long list of systemic drugs used for treatment, such as systemic corticosteroids, thalidomide, levamisole, dapsone, pentoxifylline, low-dose interferon-α, colchicine, and amlexanox [4]. However, the main course of treatment for major recurrent aphthous ulcers (RAU) is systemic corticosteroids with immuno-modulating agents. Steroids have been shown to provide symptomatic relief, whereas levamisole seems to provide symptomatic relief and alter the disease’s course. We can use systemic steroids such as prednisone and levamisole therapy to treat the ulcers associated with major RAU [5,6,7].

This paper presents a case of major recurrent aphthae where we use a combination of systemic corticosteroids and levamisole to treat the patient.

## 2. Case Report

A 17-year-old boy reported to the outpatient Department of Oral Medicine and Radiology with ulcers on his lips, tongue, and the floor of his mouth, which left scars after healing. His medical history revealed that he has been suffering from these episodes for the past 3 years, and has already been treated with various topical agents, steroids, and antibiotics, but reported no significant relief over the past year. The episodes of ulcers had increased. These lesions were extremely painful and interfered with eating, drinking, and speaking. There was no relationship with food or trauma, nor any history of allergies, but he reported flares during periods of emotional stress and during school exams. We recorded his pain on a visual analogue scale (VAS), with a score of 8.

**Figure 1:**
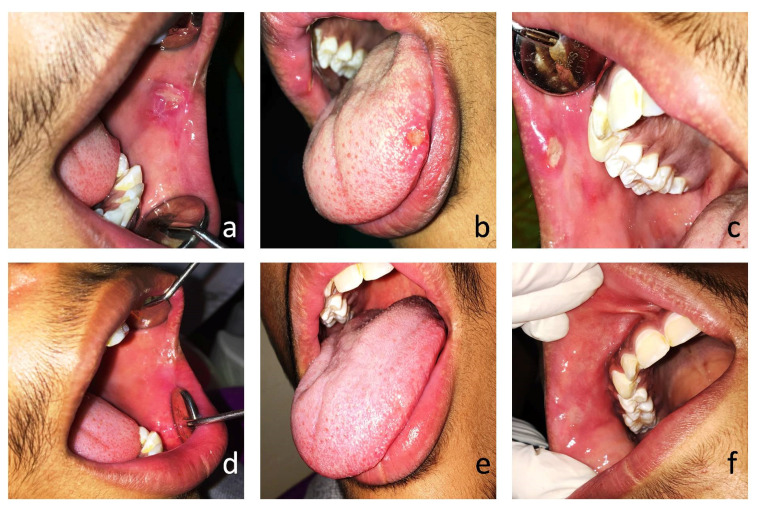
(**a**) An ulcer larger than 10 mm on the left retro commissure area; (**b**) an ulcer of10 mm in size seen on the left lateral border of the tongue; (**c**) an ulcer on the right upper labial mucosa of 10 mm in size; (**d**) a healed ulcer with scarring evident on the left retro commissure area; (**e**) a healed ulcer on the left lateral border of the tongue; and (**f**) a healed ulcer with scarring evident on the right upper labial mucosa.

Clinical examination showed an asymptomatic ulcer on the left retro commissure area with a perilesional erythematous halo, which was covered with a pseudo-membrane and was over 10 mm in diameter (Figure 1a). Ulcers with a similar presentation were seen on the tongue’s left lateral border (Figure 1b) and on the right upper labial mucosa (Figure 1c). A diagnosis of major RAU was given based on the history, symptoms, and clinical examination. No relevant medical or family history was noted. Detailed medical history was taken in order to rule out systemic disorders associated with lesions clinically similar to RAS, such as nutritional deficiencies leading to anemia, inflammatory bowel diseases, cyclic neutropenia, Behcet’s syndrome, Sweet’s syndrome, and MAGIC syndrome. There was no history of periodic fever, genital or cutaneous ulcers, pharyngitis, or cervical adenitis. Ophthalmological examination revealed no ocular ulcers. The patient came to us with his previous colonoscopy reports, and they were normal with no sign of inflammatory bowel disease. The patient did not have a history of chronic severe malnutrition, anemia, abdominal pain, or diarrhea. All laboratory investigations—including a complete blood count, erythrocyte sedimentation rate (ESR), C-reactive protein (CRP), and levels of folic acid, iron, ferritin, and vitamins B2, B6, and B12—were in the normal range.

The patient’s previous therapies included high-potency topical steroids, tacrolimus ointment, chlorhexidine rinses, topical tetracycline, topical lidocaine, oral colchicine (0.4 mg twice daily), oral azathioprine (200 mg/d), and oral prednisone ranging in doses of 10 to 40 mg. Only prednisone out of all the above showed positive results, and the dose required to ease symptoms sufficiently was 40 mg/day over the previous year. For the past year the patient was on prednisone (40 mg/day). Despite this prednisone dose, the patient was not comfortable during food intake and speech articulation, and during exams, the lesions exacerbated further. Hence, we added levamisole to his previous therapeutic regime, and the patient responded dramatically to this combination.

The patient was given systemic prednisone (15 mg per day) orally and the immune-modulating agent levamisole (50 mg tab), to be taken 3 times daily (150 mg total dose) for 3 consecutive days per week, and the regime was continued for 3 months. There was a partial regression of the lesions after the first application, and the VAS score was reduced to 3. Two weeks after the administration of the drugs, the patient showed 80% clinical improvement of ulcerations. Further applications brought about partial healing of the ulcers on the left retro commissure area (Figure 1d) and complete healing of the ulcers on the tongue (Figure 1e) and upper labial mucosa (Figure 1f). Over the next several weeks, all remaining ulcerations had healed entirely. Prednisone was stopped after tapering its dosage slowly after 1 month, and levamisole was continued for a further 3-month period. The patient was followed up for one year; there was a significant reduction in the severity, frequency, and number of ulcerations, as well as a significant reduction in pain. During this period, no adverse effects of the drug were observed.

## 3. Discussion

Recurrent aphthous ulcers (RAU), also called ‘canker’ sores, are the most common oral ulcerative condition seen in the general population. Typically, ulcers are recurrent, small ovoids covered with a yellowish pseudo-membrane and surrounded by a red halo, and occur mostly on non-keratinized mucosa. The typical healing time of ulcers is 7–14 days. RAS is divided into three different types based on size and number: minor, major, and herpetic form ulcers [8] (Table 1).

**Figure 2:**
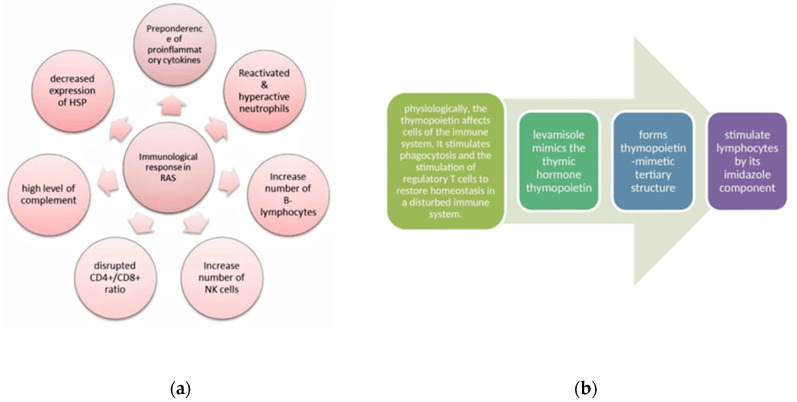
(**a**) Mechanisms of the disrupted immunologic response in RAS, and (**b**) the mechanism of levamisole action.

The etiopathogenesis of RAS is not fully understood. Various factors triggering RAS are genetic predisposition, infections (bacterial and viral), food allergies, systemic diseases (e.g., AIDS, celiac disease, and inflammatory bowel disease), vitamin deficiencies, increased oxidative stress, hormonal imbalance, and mechanical injuries. One of the vital factors that may induce and determine the type of immune response in the human body is cytokines [9]. Figure 2a shows the primary mechanism of the immunologic response disruption in RAS.

There are various theories about the etiopathogenesis and treatment of RAS, and several medications—such as corticosteroids, vitamins, antibiotics, and levamisole—have been used in the treatment of RAS. For simple cases of RAS, topical anesthetics and analgesics are used. Topical steroid creams or lotions, tetracycline suspension, and medicated toothpaste with aminoglycoside enzymes are also used [10]. Levamisole is an antihelminthic drug; it acts both as an immunosuppressant and an immune enhancer: it is an immunosuppressant at larger dosages, and an immune enhancer at smaller dosages, and has been used in clinical trials for the therapy of aphthous ulcers [11]. The standard dose of levamisole is 150 mg/day, with or without combination with steroids (15 mg Prednisone) [12].

The mechanism of levamisole action (Figure 2b) is because of its ability to restore the immune function of T lymphocytes, B lymphocytes, monocytes, and macrophages. Some patients have reported adverse effects such as flu-like symptoms, GI disturbances, insomnia, granulocytopenia, allergic manifestations, and muscle pain [13]. Owing to a wide variety of immunological effects, both in vivo and in vitro, levamisole has been used in clinical trials for RAS therapy [14]. Various studies have shown the effectiveness of levamisole in reducing ulcer size, number, duration, and frequency (Table 2).

Five methods of administration of levamisole are:

- 150 mg daily for 3 consecutive days/weeks
- 150 mg for 3 consecutive days every other week
- 50 mg 3 times daily for 2 consecutive days every week
- 150 mg three times daily for 3 consecutive days/weeks
- 150 mg daily for 3 consecutive days/weeks with an interval of 2 weeks [20].

Levamisole is an antihelminthic drug that also apparently acts as an immunosuppressant at prolonged dosages, and as an immunopotentiator at lower dosages, in RAS therapy. The standard dose of levamisole is 150 mg per day in almost every study for RAS treatment, but the treatment duration varied from 3 consecutive days per episode to 11 consecutive days [8].

Levamisole has been used to treat multiple chronic oral ulcerative lesions—such as mucous membrane pemphigoid, oral lichen planus (OLP), and pemphigus vulgaris—with varied results. In one study, all patients were given 150 mg/day of levamisole and 15 mg/day of prednisolone for 3 consecutive days each week, along with topically applied dexamethasone orobase. The addition of levamisole to prednisolone produced improved results in managing the above-stated mucocutaneous disorders [21].

The mechanism of action of steroids is a result of anti-inflammatory and immunosuppressive actions. Topical corticosteroids should be advised in moderate cases where primary methods have failed. Systemic corticosteroid therapy is recommended for patients with recalcitrant cases: hydrocortisone (20 mg), triamcinolone (4 mg), or prednisone (10–30 mg/day) for 10–15 days [13].

When the disease is not adequately controlled with oral corticosteroids, immunomodulators are the drug of choice for reducing the severity of the outbreak and preventing further outbreaks. A recent study explored the effects of daily ascorbic acid (2000 mg/day) in managing RAS. Ascorbate decreases neutrophil-mediated inflammation via the modulation of reactive oxygen species. Ascorbic acid should be considered in RAS treatment because of its low side effect profile [22]. Low-level laser therapy at a wavelength of 658 nm may also benefit RAS patients as an adjunctive, as it was equal to pharmacological treatment in managing pain and inflammation and increasing the re-epithelization of aphthous ulcers [23].

Many systemic disorders are associated with lesions clinically similar to RAS, such as nutritional deficiencies leading to anemia, inflammatory bowel diseases, cyclic neutropenia, Behcet’s syndrome, PFAPA, Sweet’s syndrome, and MAGIC syndrome [1]. RAS-like lesions may be an early sign of systemic autoimmune disease, as they are associated with an increased risk of the development of autoimmune diseases. A recent study by Lee CY suggested that the overall risk of autoimmune disease development is significantly greater in RAS patients. Patients with RAS showed an increased risk of Behcet’s disease, systemic lupus erythematous, ankylosing spondylitis, Hashimoto’s thyroiditis, Graves’ disease, and rheumatoid arthritis. Thus, proper medical and family history, as well as a detailed clinical examination of the patient, is necessary in order to rule out any underlying systemic cause [24].

Because of the unclear pathophysiology and etiology of RAS, most of the treatment given is symptomatic. Literature shows that recurrent severe aphthous ulcers are best treated with levamisole accompanied with systemic steroids, as they reduce healing time, alleviate the pain, decrease the number and size of ulcers, and halt recurrence.

## 4. Conclusions

To sum up, we treated a young patient with severe, debilitating major recurrent aphthous ulcers resistant to multiple standard therapies. The patient’s lesions responded dramatically after the addition of levamisole to his previous therapeutic regime. In this case, the treatment modality gave significant relief to the patient from symptoms and decreased the recurrence rate. There was a partial regression of the lesions after the first application, and further applications brought about partial healing of all the ulcers, while over the next several weeks all remaining ulcerations healed entirely. The patient was followed-up for one year; there was a significant reduction in the severity, frequency, and pain of ulcerations. Levamisole given along with steroids shows promising results in treating patients with major aphthous ulcers, with negligible side effects. Moreover, the addition of levamisole to steroids can significantly reduce the dose of steroid required to control such conditions, reducing the side effects of the steroids.

## Figures and Tables

**Table 1:** Types of RAS with clinical features.

Character	Minor RAS	Major RAS	Herpetiform Ulcers
Peak age (decade)	2nd	1st and 2nd	3rd
Number of ulcers	1–5	1–3	5–20 (up to 100)
Size of ulcers (mm)	Less than 10 mm	More than 10 mm	1–2 mm
Duration	7–14 days	2 weeks–3 months	7–14 days
Healing with scarring	No	Yes	No
Site	Non-keratinized mucosa—especially labial/buccal mucosa; dorsum and lateral borders of the tongue	Keratinized and non-keratinized mucosa, particularly the soft palate	Non-keratinized Mucosa, but Particularly the floor of the mouth and the ventral surface of the tongue
Percentage of cases	85% of all cases	10–15% of all cases	5–10% of all cases

**Table 2:** Studies showing the effectiveness of levamisole for RAS.

Author	Year	Index of Improvement
J. Symoens [15]	1974	Reduction in pain, number, frequency
J DE Meyer et al. [16]	1977	Reduction in number, frequency and duration
Van de Heyning [17]	1978	Reduction in pain, number, duration
Weckx et al. [18]	2009	Reduction in size, number, frequency and duration
Picciani BLS [5]	2010	Reduction in number, frequency and duration
Sharda et al. [19]	2014	Reduction in pain, number, frequency and duration
Bathina P et al. [10]	2015	Reduction in pain, number, duration
TAR Raja [11]	2015	Reduction in pain, number, frequency

## Data Availability

Data is contained within the article.
